# A comprehensive assessment of physical image quality of five different scanners for head CT imaging as clinically used at a single hospital centre—A phantom study

**DOI:** 10.1371/journal.pone.0245374

**Published:** 2021-01-14

**Authors:** Patrizio Barca, Fabio Paolicchi, Giacomo Aringhieri, Federica Palmas, Daniela Marfisi, Maria Evelina Fantacci, Davide Caramella, Marco Giannelli

**Affiliations:** 1 Unit of Medical Physics, Pisa University Hospital “Azienda Ospedaliero-Universitaria Pisana”, Pisa, Italy; 2 Diagnostic and Interventional Radiology, University of Pisa, Pisa, Italy; 3 Department of Physics, University of Pisa, Pisa, Italy; 4 INFN, Pisa section, Pisa, Italy; Stanford University School of Medicine, UNITED STATES

## Abstract

Nowadays, given the technological advance in CT imaging and increasing heterogeneity in characteristics of CT scanners, a number of CT scanners with different manufacturers/technologies are often installed in a hospital centre and used by various departments. In this phantom study, a comprehensive assessment of image quality of 5 scanners (from 3 manufacturers and with different models) for head CT imaging, as clinically used at a single hospital centre, was hence carried out. Helical and/or sequential acquisitions of the Catphan-504 phantom were performed, using the scanning protocols (CTDI_vol_ range: 54.7–57.5 mGy) employed by the staff of various Radiology/Neuroradiology departments of our institution for routine head examinations. CT image quality for each scanner/acquisition protocol was assessed through noise level, noise power spectrum (NPS), contrast-to-noise ratio (CNR), modulation transfer function (MTF), low contrast detectability (LCD) and non-uniformity index analyses. Noise values ranged from 3.5 HU to 5.7 HU across scanners/acquisition protocols. NPS curves differed in terms of peak position (range: 0.21–0.30 mm^-1^). A substantial variation of CNR values with scanner/acquisition protocol was observed for different contrast inserts. The coefficient of variation (standard deviation divided by mean value) of CNR values across scanners/acquisition protocols was 18.3%, 31.4%, 34.2%, 30.4% and 30% for teflon, delrin, LDPE, polystyrene and acrylic insert, respectively. An appreciable difference in MTF curves across scanners/acquisition protocols was revealed, with a coefficient of variation of f_50%_/f_10%_ of MTF curves across scanners/acquisition protocols of 10.1%/7.4%. A relevant difference in LCD performance of different scanners/acquisition protocols was found. The range of contrast threshold for a typical object size of 3 mm was 3.7–5.8 HU. Moreover, appreciable differences in terms of NUI values (range: 4.1%-8.3%) were found. The analysis of several quality indices showed a non-negligible variability in head CT imaging capabilities across different scanners/acquisition protocols. This highlights the importance of a physical in-depth characterization of image quality for each CT scanner as clinically used, in order to optimize CT imaging procedures.

## 1. Introduction

In recent years, the extraordinary technical advances in x-ray computed tomography (CT) have largely increased its use in the clinical practice. Thus, CT has become a fundamental imaging tool in several body as well as head applications, providing useful information for diagnosis and patient care [1–3].

Given that CT imaging represents the largest source of population exposure to ionizing radiation in industrialized countries [4, 5] and increased radiation dose may increase the risk of cancer [6], it is important to minimize radiation dose (without compromising the diagnostic potential) through an optimization and standardization of acquisition protocols [7–9]. Accordingly, various technical approaches (e.g. tube current modulation, automatic exposure control, iterative reconstruction algorithms) to optimize CT acquisitions in various applications can be used [10–15]. Furthermore, some previous studies have described phantom-based methods to optimize acquisition protocol for specific CT imaging techniques and applications, exploiting various physical image quality indices [9, 16–21]. For instance, Zhang et al [9] have developed a CT protocol optimization platform by combining task-based detectability calculations with a graphical user interface that demonstrates the trade-off between dose and image quality. Their platform can be used to improve individual dose efficiency and acquisition protocol consistency across various patient sizes and CT scanners. Berta et al [19] have described a method to objectively evaluate image quality when new clinical protocol performances must be compared with a standard reference. This quantitative approach has been applied to the images of a typical routine abdominal protocol, which were reconstructed with the standard filtered back projection (FBP) and the Iterative Reconstruction in Image Space (IRIS) algorithm. An adaptable and global approach for optimizing CT protocols, by evaluating the influence of acquisition parameters and iterative reconstruction algorithms, has been proposed and implemented in a software program by Greffier et al [20]. Moreover, Noferini et al [21] have proposed and validated a method that employs a Channelling Hotelling model Observer in a CT protocol optimization program, with the aim at assuring that scanners are working at their own best with regard to image quality and patient exposure.

Nonetheless, an objective and in-depth physical characterization of performance of CT scanners [22–29], acquisition methods [30–35] and image reconstruction algorithms [36–41], in terms of specific quantitative image quality indices, remains an essential step. Indeed, these indices have the potential to serve as a basis for guiding and optimizing clinical protocols [42–44]. In this regard, previous phantom studies have shown that CT image quality can vary substantially when acquisitions are performed on different scanners [45–48], even using similar acquisition protocols [49–52]. Therefore, a careful assessment of image quality for each specific CT imaging technique and application is recommended.

Given also time efficiency and cost considerations, head CT is a first line imaging examination for assessing neurological disorders [53–55]. In particular, head CT imaging is highly sensitive to bleeding, and is an essential diagnostic modality to investigate osseous structures as well as to detect calcifications. Moreover, it is usually preferred to magnetic resonance imaging for its wide availability, rapid acquisition and high spatial resolution [56, 57]. In this regard, 5 CT scanners, with different technical characteristics, are currently installed at our hospital centre. It should be noted that radiologists and neuroradiologists by various departments of our institution execute head CT examinations on these scanners by using different acquisition protocols, with similar radiation dose values. Therefore, toward an optimization of CT imaging procedures at our hospital centre, the aim of this phantom study was to comprehensively assess whether and how head CT physical image quality varies with different scanners as clinically used for routine examinations.

## 2. Materials and methods

### 2.1. Scanners and phantom acquisitions

**Fig 1:**
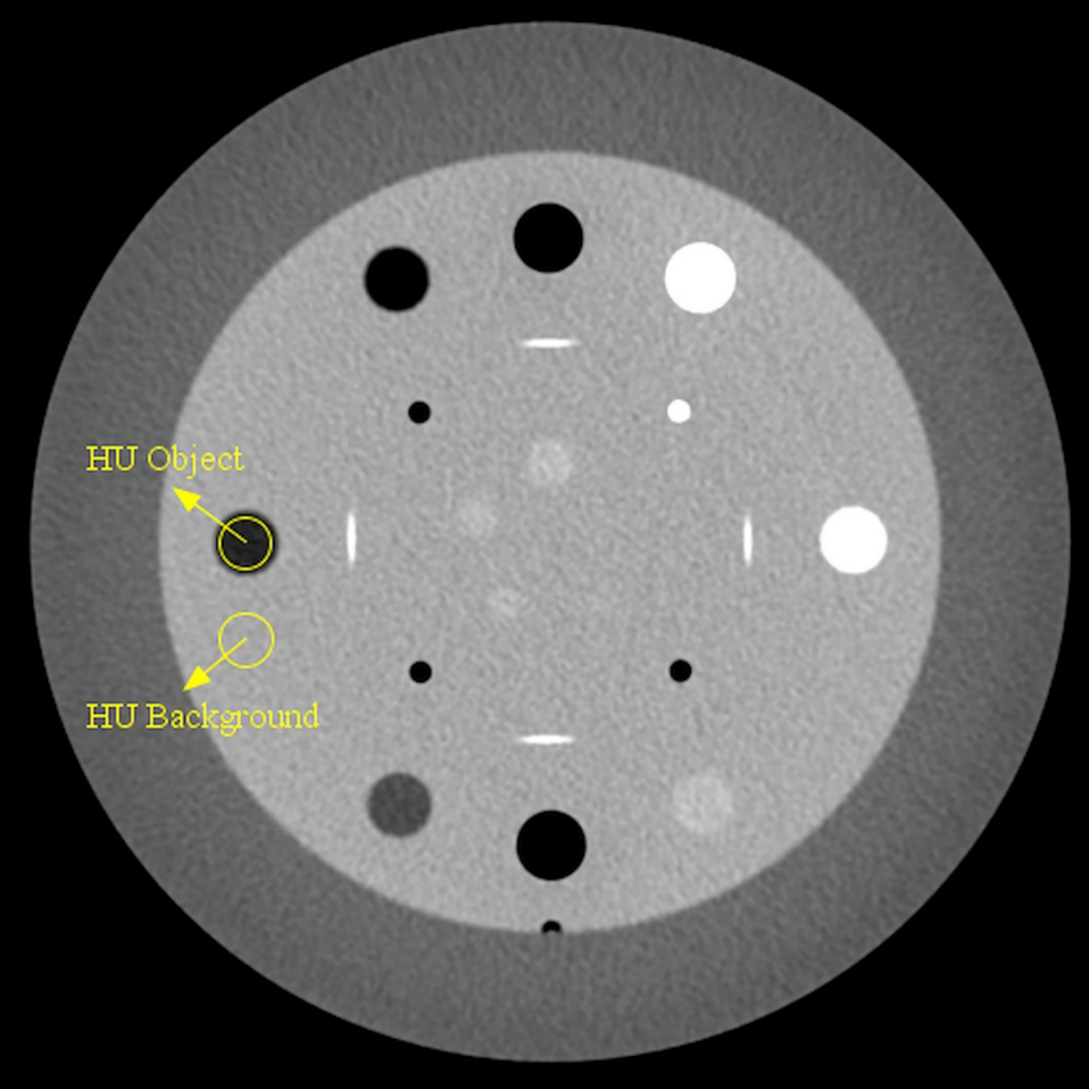
ROIs position for CNR evaluation in the CTP404 module of the Catphan-504 phantom. The depicted ROIs refer to the LDPE contrast insert.

Images of the Catphan-504 phantom (The Phantom Laboratory, NY, USA) were acquired on 5 CT scanners (one 128-slice, two 64-slice and two 16-slice CT scanners) from different manufacturers (Table 1). The phantom has a cylindrical shape with a diameter of 20 cm and it is composed of four modules. In particular, the CTP486 and CTP404 modules of the phantom were employed for CT acquisitions. The CTP486 module is a homogeneous water-equivalent module, while the CTP404 module includes multiple inserts of different materials in a water-equivalent background (Fig 1). Nominal CT Hounsfield units (HU) values of the inserts are reported in Table 2.

**Table 1:** CT scanners enrolled in the study.

Scanner ID	Manufacturer	Model	Number of slices
Toshiba-16	Toshiba Medical Systems, Japan	Aquilion 16	16
GE-16RT	GE Healthcare, USA	LightSpeed RT16	16
GE-64VCT	GE Healthcare, USA	LightSpeed VCT	64
Siemens-64	Siemens Healthineers, Germany	Sensation 64	64
GE-128	GE Healthcare, USA	Discovery 750 HD	128

**Table 2:** Nominal HU values of the Catphan-CTP404 module inserts [58].

Material	HU range (reference values)
LDPE	-121: -87
Polystyrene	-65: -29
Acrylic	92: 137
Delrin	344: 387
Teflon	941: 1060

Sequential and/or helical clinical CT head protocols, with different acquisition parameters but similar radiation doses in terms of CTDI_vol_, were employed for acquisitions on CT scanners as indicated in Table 3. CT images were reconstructed by using the conventional filtered back-projection algorithm.

**Table 3:** Clinical head CT imaging protocols used for each scanner by the staff of various Radiology/Neuroradiology departments at the Pisa university hospital.

Scanner ID	Scan mode	Tube load (mAs)	Tube voltage (kVp)	Pitch factor	Slice thickness (mm)	S-FOV	Collimation (mm)	CTDI_vol_ (mGy)	Reconstruction kernel
Toshiba-16	h	165	120	0.688	4	M	16	56.6	FC64
	s	220	120	-	4	M	8	54.7	FC64
GE-16RT	s	330	120	-	2.5	head	10	57	Standard
GE-64VCT	s	320	120	-	5	head	20	57.5	Standard
Siemens-64	s	380	120	-	6	head	18	55.7	H31S
GE-128	h	280	120	0.969	5	head	20	56.1	Standard
	s	280	120	-	5	head	20	55.9	Standard

h = helical, s = sequential.

### 2.2. Physical image quality assessment

For each CT scanner/acquisition protocol, physical image quality was assessed through a number of indices which are strictly related to the main characteristics of images in terms of noise, spatial resolution and contrast properties [59–64]. In particular, quantitative metrics of noise level, noise power spectrum (NPS), contrast-to-noise ratio (CNR), modulation transfer function (MTF), low contrast detectability (LCD) and non-uniformity index (NUI) were estimated.

For each image quality index and CT scanner/acquisition protocol, 5 repeated acquisitions were performed. The estimated value and uncertainty of a quality index were obtained as the mean value and standard deviation (SD) across repeated measurements, respectively.

Image analysis was performed by using ImageJ (Wayne Rasband, National Institute of Health, USA), Origin (OriginLab Corporation, MA, USA) and Matlab (The MathWorks, Inc., MA, USA) software packages.

### 2.2.1. Noise level

Noise level was evaluated by computing the SD of HU values within a 4.5 cm diameter circular region of interest (ROI), placed at the centre of the acquisition slab central image of the uniform CTP486 module.

### 2.2.2. Noise power spectrum (NPS)

Texture properties of CT image noise were assessed by computing the NPS (i.e. the spatial frequency distribution of noise) [65, 66]:

$$NPS\left(f_{x},f_{y}\right)=\frac{\Delta x\cdot \Delta y}{N_{x}\cdot N_{y}}\cdot <{\left|FFT\left(ROI_{noise}\right)\right|}^{2}>$$ (1)

where f_x_/f_y_ are the spatial frequencies along the main orthogonal directions, Δx/Δy are the voxel sizes, N_x_/N_y_ are the number of voxels for each direction, FFT is the two-dimensional (2D) fast Fourier transform, ROI_noise_(x,y) is the local value of an "only-noise" ROI and < > indicates the ensemble average (i.e. the average across measurements performed on a number of ROIs). In particular, for each acquisition, an ensemble of 5 ROIs (64 pixels × 64 pixels) was selected from the acquisition slab central image of the uniform CTP486 phantom module. Given the radial symmetry of the 2D NPS, radial profiles along many directions were averaged in order to obtain the one dimensional NPS curve. In particular, the selection of radial profiles was carried out every 10° over 360°, obtaining a total of 36 radial profiles.

In order to estimate peak position, NPS curves were fitted by using a specific peak function (namely "InvsPoly") implemented in Origin:

$$f(x)=y_{0}+\frac{A}{1+A_{1}{\left(2\frac{x-x_{c}}{w}\right)}^{2}+A_{2}{\left(2\frac{x-x_{c}}{w}\right)}^{4}+A_{3}{\left(2\frac{x-x_{c}}{w}\right)}^{6}}$$ (2)

where x_c_ is the peak position, A/A_1_/A_2_/A_3_ are coefficients related to the amplitude of the peak, w is a parameter related to the width of the curve and y_0_ is an offset.

### 2.2.3. Contrast-to-noise ratio (CNR)

Images of different inserts (i.e. teflon, delrin, LDPE, polystyrene, acrylic) of the CTP404 phantom module, whose nominal HU values are reported in Table 2 [60], were used to estimate CNR. In particular, CNR was estimated as follows [67, 68]:

$$CNR=\frac{\left|HU_{obj}-HU_{bkg}\right|}{\sigma_{bkg}}$$ (3)

where HU_obj_ and HU_bkg_ are the mean of HU values in a circular ROI (diameter 9 mm) in the considered insert and background region, respectively, while σ_bkg_ is the SD of CT numbers in a background region close to the considered insert (Fig 1).

### 2.2.4. Modulation transfer function (MTF)

Spatial resolution was evaluated in the spatial frequency domain. The modulation transfer function (MTF) was computed through the circular edge method (i.e. starting from edge spread function measurements) as follows [65, 69]:

$$MTF\left(f\right)=\frac{\left|\int \frac{d}{dx}ESF\left(x\right)\cdot e^{-i2\pi fx}dx\right|}{\int \frac{d}{dx}ESF\left(x\right)dx}$$ (4)

where f and ESF represent the spatial frequency and edge spread function, respectively. In particular, ESF was referred to the teflon insert of the CTP404 phantom module [58]. The spatial frequencies corresponding to 50% (f_50%_) and 10% (f_10%_) of each MTF curve were estimated.

### 2.2.5. Low contrast detectability (LCD)

A low-contrast detectability (LCD) analysis was performed on the homogeneous module of the phantom (CTP486), using a statistical method [64, 70, 71] based on the Rose model of threshold signal detectability [72–75]. Specifically, squared ROIs ranging from 2 × 2 pixels to 11 × 11 pixels (i.e. object sizes approximately ranging from 0.85 mm to 4.75 mm) were employed in this analysis. A set of 900 ROIs (placed randomly and covering all phantom image) were analysed for each ROI size and the contrast threshold (C_t_) was evaluated, by assuming a Gaussian distribution of the average of HU values within each ROI [64, 70, 71], as follows:

$$C_{t}\left(HU\right)=3.29\cdot \Delta$$ (5)

where Δ is the standard deviation of the Gaussian distribution, with mean value μ. Accordingly, a low contrast object of the same size as the ROIs can be revealed at a confidence level of 95% if its mean HU value differs more than 3.29 Δ from μ [76].

### 2.2.6. Non-uniformity index (NUI)

Variation of CT numbers within acquisition slab central image of the uniform CTP486 module was assessed through the non-uniformity index (NUI). In particular, NUI was estimated by adapting the method proposed by Li et al and suggested by the AAPM TG233 report for assessing spatial non-uniformity of noise maps [38, 77]. Images of the entire phantom were divided into M = 249 small ROIs of 7 mm × 7 mm size. Then, NUI index was calculated as:

$$NUI(\%)=\frac{100}{I}\sqrt{\frac{1}{M-1}\sum_{j=1}^{M}{\left(I_{j}-I\right)}^{2}}$$ (6)

where I_j_ and <I> are the average of CT numbers within the j-th ROI and average of all I_j_ values, respectively.

## 3. Results

Noise results are reported in Table 4. An appreciable difference in noise values across different scanners/acquisition protocols was found. Noise values ranged from 3.5 ± 0.1 HU (Siemens-64 scanner) to 5.7 ± 0.1 HU (GE-16RT scanner).

**Table 4:** Noise (σ) values (mean ± standard deviation across five repeated measurements), for each CT scanner/acquisition protocol.

Scanner ID	Scan mode	σ (HU)
Toshiba-16	h	4.2 ± 0.1
	s	4.6 ± 0.2
GE-16RT	s	5.7 ± 0.1
GE-64VCT	s	3.9 ± 0.1
Siemens-64	s	3.5 ± 0.1
GE-128	h	4.6 ± 0.1
	s	4.3 ± 0.1

h = helical, s = sequential.

**Fig 2:**
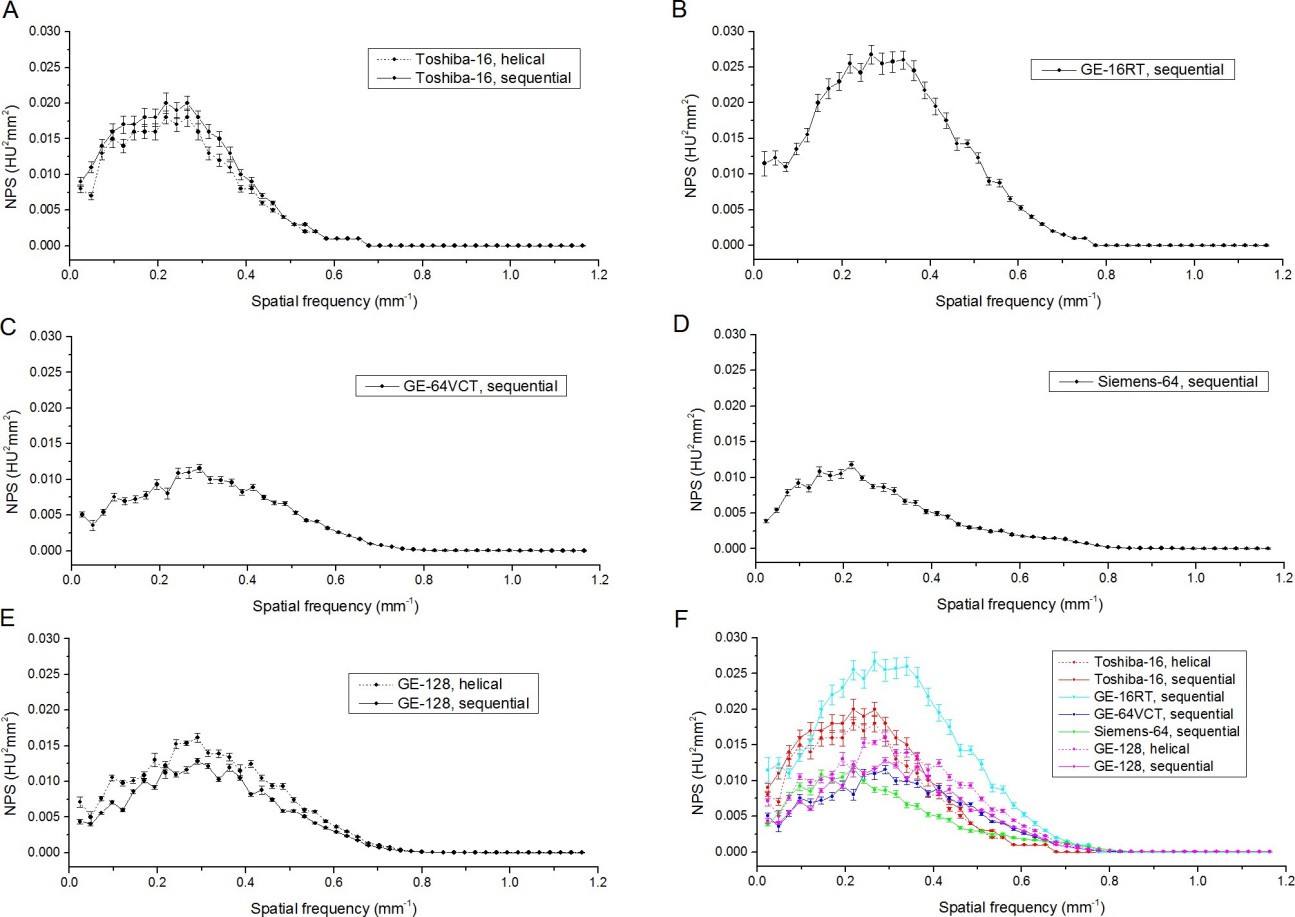
Noise power spectrum (NPS) of images acquired using different CT scanners/acquisitions protocols. Panels A, B, C, D and E show the NPS curves (mean ± standard deviation across five repeated measurements) for Toshiba-16, GE-16RT, GE-64VCT, Siemens-64 and GE-128 scanners, respectively. In order to better compare the different NPS curves, panel F shows on the same graphic the NPS curves for all scanners/acquisition protocols.

Fig 2 shows NPS curves for each scanner/acquisition protocol. NPS curves differed in terms of peak position (Table 5), with Toshiba-16 (both axial and helical scan mode) (~ 0.22 mm^-1^) and Siemens-64 (~ 0.21 mm^-1^) scanners showing lower peak position with respect to the other scanners (> 0.29 mm^-1^).

**Table 5:** Peak position of NPS curves for each CT scanner/acquisition protocol.

Scanner ID	Scan mode	Peak position (mm^-1^)
Toshiba-16	h	0.22 ± 0.01
	s	0.22 ± 0.01
GE-16RT	s	0.29 ± 0.01
GE-64VCT	s	0.29 ± 0.01
Siemens-64	s	0.21 ± 0.01
GE-128	h	0.30 ± 0.01
	s	0.30 ± 0.01

h = helical, s = sequential.

CNR results are reported in detail in Table 6. A substantial variation of CNR values with scanner/acquisition protocol was observed for all inserts (teflon, delrin, LDPE, polystyrene, acrylic). The coefficient of variation (standard deviation divided by mean value) of CNR values across different scanners/acquisition protocols was 18.3%, 31.4%, 34.2%, 30.4% and 30% for teflon, delrin, LDPE, polystyrene and acrylic insert, respectively.

**Table 6:** CNR values (mean ± standard deviation across five repeated measurements) of different inserts from the CTP404 phantom, for each CT scanner/acquisition protocol.

Scanner ID	Scan mode	Teflon	Delrin	LDPE	Polystyrene	Acrylic
Toshiba-16	h	167 ± 6	44 ± 3	43 ± 3	31 ± 1	5.5 ± 0.6
	s	150 ± 20	38 ± 2	42 ± 4	28 ± 2	5.3 ± 0.5
GE-16RT	s	140 ± 11	41 ± 2	35 ± 2	23 ± 2	4.7 ± 0.5
GE-64VCT	s	209 ± 20	65 ± 8	54 ± 4	38 ± 4	7.4 ± 0.4
Siemens-64	s	233 ± 22	89 ± 6	73 ± 5	55 ± 2	10.6 ± 0.8
GE-128	h	200 ± 10	59 ± 3	31 ± 2	33 ± 1	6.2 ± 0.4
	s	177 ± 12	64 ± 5	31 ± 3	30 ± 3	6.4 ± 0.5

h = helical, s = sequential.

**Fig 3:**
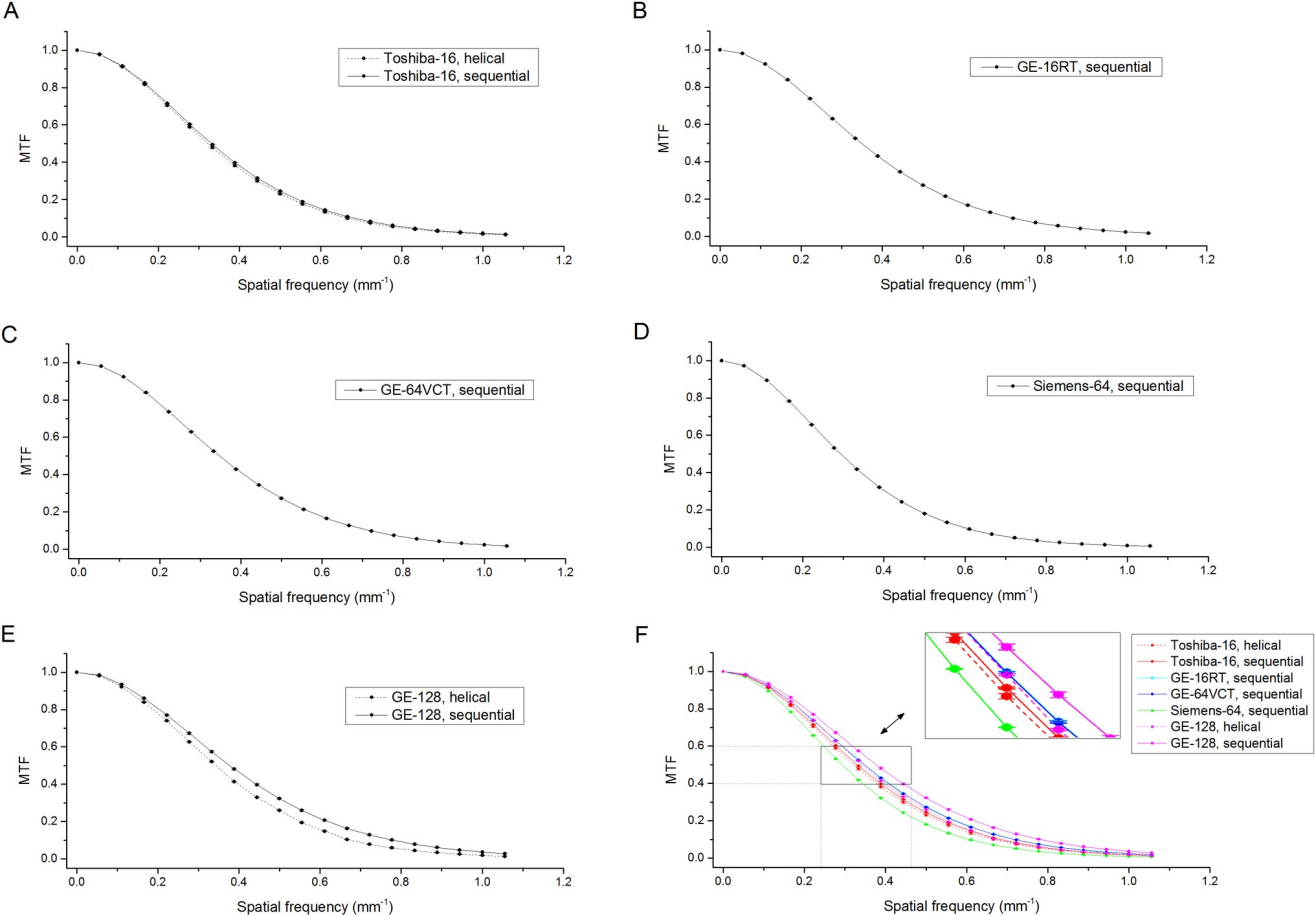
Modulation transfer function (MTF) curves of images acquired using different CT scanners/acquisitions protocols. Panels A, B, C, D and E show the MTF curves (mean ± standard deviation across five repeated measurements) for Toshiba-16, GE-16RT, GE-64VCT, Siemens-64 and GE-128 scanners, respectively. In order to better compare the different MTF curves, Panel F shows on the same graphic the MTF curves for all scanners/acquisition protocols, with a zoomed in version for the 40%-60% range of MTF.

MTF results are shown in Fig 3 and Table 7. MTF curves varied appreciably across scanners/acquisition protocols. Overall, GE-128, GE-64VCT and GE-16RT scanners were characterized by MTF curves with higher values with respect to the other scanners (Fig 3F). Specifically, GE-128 scanner with sequential acquisition protocol showed the best performance in terms of spatial resolution properties. The coefficient of variationof f_50%_ and f_10%_ across scanners/acquisition protocols was 10.1% and 7.4%, respectively.

**Table 7:** Spatial frequencies (mean ± standard deviation across five repeated measurements) corresponding to 50% (f_50%_) and 10% (f_10%_) of the MTF curves, for each CT scanner/acquisition protocol.

Scanner ID	Scan mode	f_50%_ (mm^-1^)	f_10%_ (mm^-1^)
Toshiba-16	h	0.31 ± 0.01	0.66 ± 0.02
	s	0.31 ± 0.01	0.66 ± 0.02
GE-16RT	s	0.35 ± 0.01	0.65 ± 0.03
GE-64VCT	s	0.35 ± 0.01	0.65 ± 0.02
Siemens-64	s	0.28 ± 0.02	0.63 ± 0.03
GE-128	h	0.34 ± 0.01	0.67 ± 0.02
	s	0.38 ± 0.01	0.78 ± 0.02

h = helical, s = sequential.

**Fig 4:**
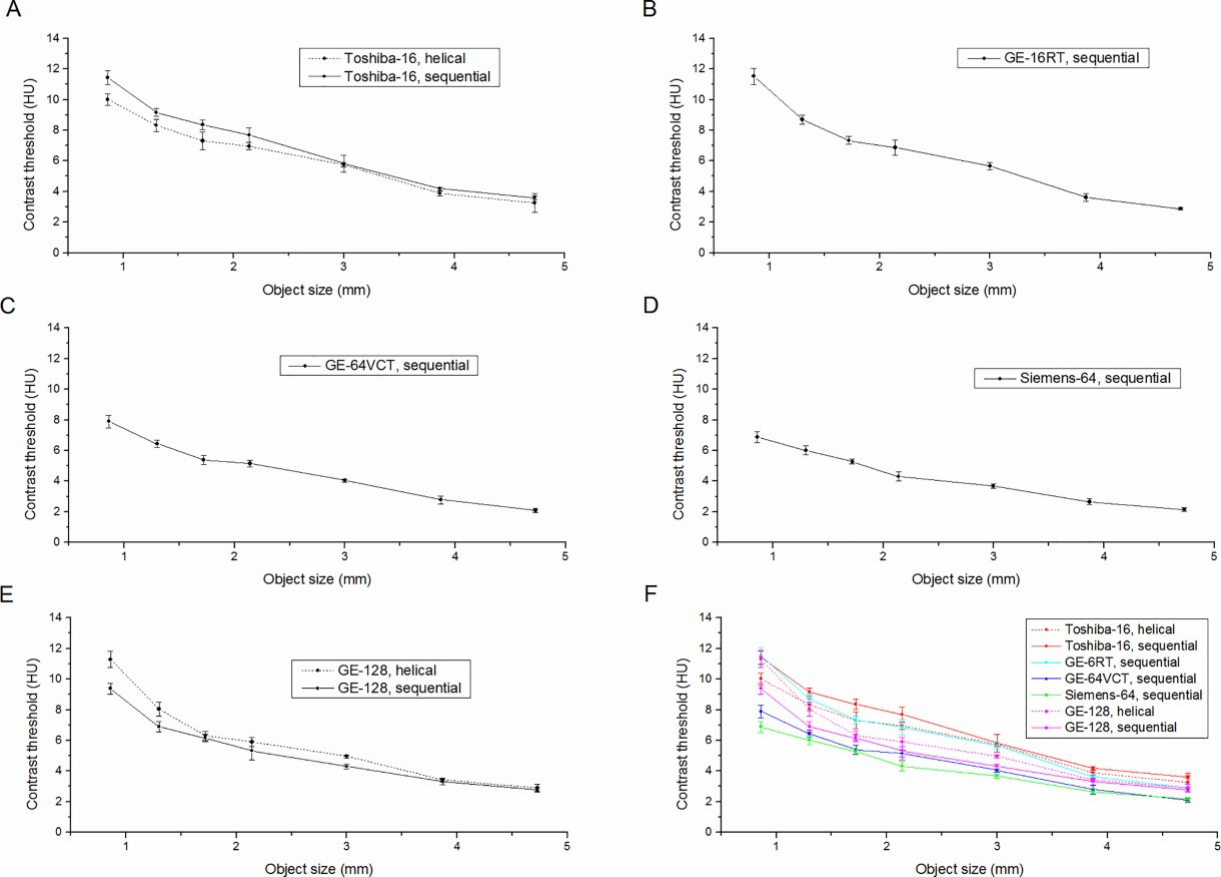
Low-contrast detectability (LCD) curves of images acquired using different CT scanners/acquisition protocols. Panels A, B, C, D and E show the LCD curves (mean ± standard deviation across five repeated measurements) for Toshiba-16, GE-16RT, GE-64VCT, Siemens-64 and GE-128 scanners, respectively. In order to better compare the different LCD curves, Panel F shows on the same graphic the LCD curves for all scanners/acquisition protocols.

A relevant difference in LCD performance of different scanners/acquisition protocols was found (Fig 4). For all object sizes, the Siemens-64 and GE-64VCT scanners showed lower contrast threshold values with respect to the other scanners. The contrast threshold for a typical object size of 3 mm ranged from 3.7 HU (Siemens-64 scanner) to 5.8 HU (Toshiba-16 scanner, sequential scan mode).

NUI results are reported in detail in Table 8. NUI values ranged from 4.1% (GE-128 scanner, sequential scan mode) to 8.3% (Toshiba-16 scanner, sequential scan mode) across different CT scanners/acquisition protocols.

**Table 8:** NUI values (mean value ± standard deviation across five repeated measurements) for each CT scanner/acquisition protocol.

Scanner ID	Scan mode	NUI (%)
Toshiba-16	h	7.6 ± 0.2
	s	8.3 ± 0.3
GE-16RT	s	7.5 ± 0.3
GE-64VCT	s	8.0 ± 0.2
Siemens-64	s	4.2 ± 0.1
GE-128	h	4.2 ± 0.2
	s	4.1 ± 0.3

h = helical, s = sequential.

## 4. Discussion

Nowadays, the technological advance in CT imaging has brought increasing number of clinical exams/applications, as well as increasing heterogeneity in characteristics/performance of CT scanners. Therefore, a number of CT scanners with different models or by different manufacturers are often installed in a hospital centre and used by various departments. Previous phantom studies, mainly focused on body applications, have shown that CT image quality can vary substantially across scanners [45–48], even when similar acquisition protocols are employed [49–52]. For instance, in a multicentre study, Racine et al [45] have compared the image quality of 68 scanners, in terms of only low contrast detectability, using local clinical acquisition protocols for abdominal CT examinations. They have found an important difference in image quality levels, associated with a variability in CTDI_vol_ values, which increased with growing phantom size. Kuo et al [47] have aimed at characterizing CT practices and performance of 16 CT scanners, in terms of noise and spatial resolution (i.e. MTF), in different centres for cystic fibrosis. A large variety in CT protocols, image quality and radiation dose among the centres was found. A task-based image quality assessment of 4 “older” (model released between 2003 and 2007) and 4 “newer” (model released between 2012 and 2014) CT scanners has been performed by another study [49], using similar acquisition protocols with fixed CTDI_vol_. The authors have revealed an appreciable difference in high contrast spatial resolution and low contrast detectability across CT scanners. In the study by Zhang et al [50], both subjective and objective methods were used to evaluate the high contrast spatial resolution capabilities of three 64-slice CT scanners, using the same scanning parameters. The CT scanners exhibited different performances, which resulted more relevant for the subjective than the objective method for spatial resolution assessment. Solomon et al [52], using comparable acquisition protocols, have carried out a quantitative comparison of noise texture properties (i.e. NPS analysis) of two CT scanners, for a number of reconstruction filters. The peak frequency values ranged from 0.39 mm^-1^ to 1.03 mm^-1^ for one scanner and from 0.43 mm^-1^ to 0.62 mm^-1^ for the other.

To the best of our knowledge, this is the first phantom study which comprehensively assessed physical image quality of 5 different scanners (from various manufacturers) for head CT imaging at a single centre, considering the clinical acquisition protocols used by the staff of various Radiology/Neuroradiology departments of our hospital centre for routine examinations. Specifically, we performed an in-depth analysis of head CT image by using a number of quality indices such as noise level, NPS, CNR, MTF, LCD and NUI. Indeed, noise is one of the main factors affecting image quality, given that it can yield fluctuations in raw data and CT numbers. Noise level is usually estimated as the standard deviation of CT numbers within a relatively small ROI. However, this approach does not consider any spatial relationship among fluctuations of CT numbers within the image. We have hence included, in our study, the analysis of NPS, which is mathematically defined as the Fourier transform of the autocorrelation function and provides information regarding the correlation of the fluctuations that occur at different positions on the image [38, 65, 67, 76, 78]. NPS is strictly related to the image appearance, i.e. image texture. Notably, different CT scanners/acquisition protocols can be characterized by different image textures, even at fixed noise level [66]. Moreover, noise can affect the detectability of an object, which depends on its contrast, as well as on its size. Therefore, for various contrast objects (teflon, delrin, LDPE, polystyrene, acrilic), we assessed CNR as the ratio between contrast (i.e. difference in CT numbers between the object and background) and noise [51, 67, 68, 79]. Furthermore, we included the LCD analysis, which allows to assess the limiting detectable contrast threshold for a given object size [71, 80–83]. In addition, the spatial resolution of CT scanner systems with varying spatial frequencies was evaluated by computing MTF, defined as the Fourier transform of the point spread function of the system, which represents the modulation/loss of contrast as a function of the spatial frequency due to the limited spatial resolution of the imaging system [61]. Therefore, MTF is a descriptive metric of the CT system performance in detecting objects with decreasing sizes (related to the inverse of spatial frequency of MTF). Moreover, in order to make our analysis more complete, we performed also the NUI analysis. NUI assesses the non-uniformity degree of CT numbers within the image, which can reflect the presence of potential artifacts (e.g. beam hardening).

In line with the findings of previous studies [45–52], we revealed appreciable differences in all CT image quality indices across scanners/acquisitions protocols. Noise level (Table 4), peak position of NPS curve (Table 5), f_50%_ (Table 7), f_10%_ (Table 7) and contrast threshold for a typical object size of 3 mm (Fig 4) differed across scanners/acquisition protocols up to 62.8%, 42.8%, 35.7%, 23.8% and 56.7%, respectively. Moreover, CNR values (Table 6) varied across CT scanners/acquisition protocols, with this effect resulting lower for the high contrast teflon insert as compared to the other contrast inserts. In particular, CNR values varied across CT scanners/acquisitions protocols up to 66.4%, 134.2%, 135.5%, 139.1% and 125.5% for teflon, delrin, LDPE, polystyrene and acrylic inserts, respectively. In addition, NUI values (Table 8) differed across scanners/acquisition protocols more than 100%.

While the head CT imaging acquisition protocols for the 5 scanners at our hospital centre were characterized by similar CTDI_vol_ values (range: 54.7–57.5 mGy), there are some differences in various elements which include collimation (range: 8–20 mm), slice thickness (range: 2.5–6 mm) and reconstruction kernel (see Table 3). These differences can partly explain some results of noise, CNR and spatial resolution analyses [42, 52, 84, 85]. For instance, the relatively high slice thickness (6 mm) and soft reconstruction kernel (H31S) for acquisitions on the Siemens-64 scanner can contribute to the lower/higher noise/CNR values as well as to the lower spatial resolution properties with respect to the other scanners/acquisition protocols. Nonetheless, we cannot exclude a possible effect of scanner technology, as well as of some specific components of scanner hardware such as detectors and x-ray tube.

In the 5 CT scanners (from different manufacturers) enrolled in this study, given also the specific way of operating and needs of various Radiology/Neuroradiology departments of our hospital centre, different acquisition protocols for routine head CT examinations are employed, albeit they present similar CTDI_vol_ values. We revealed a not negligible difference in CT image quality across scanners/acquisition protocols. As future research, our results can be useful to guide an optimization of head acquisition protocols for each CT scanner [42–44] or to possibly homogenize CT image quality across scanners. Nonetheless, given that a “best” or “worst” CT scanner/acquisition protocol for all image quality analyses was not found, the characterization that we performed can be potentially employed to allow a more appropriate selection of a CT scanner/acquisition protocol for a specific clinical situation. In fact, different clinical applications may require specific image quality properties. For instance, higher performance of a CT scanner in terms of lower noise level are needed to optimize the detection of small haemorrhagic lesions. On the other hand, higher spatial resolution properties can improve metastatic brain lesions detection, while higher low contrast detectability properties are essential for a better interpretation of images acquired after ischemic stroke [86].

## 5. Conclusions

In this phantom study, a comprehensive assessment of image quality of 5 scanners (from various manufacturers and with different models) for head CT imaging, as clinically used at a single hospital centre, was performed. While similar clinical acquisition protocols in terms of dose value (i.e. CTDI_vol_) were employed, the analysis of several quality indices (including noise level, NPS, CNR, MTF, LCD and NUI) has shown an appreciable and non-negligible variability in head CT imaging capabilities across different scanners/acquisition protocols. This highlights the importance of a physical characterization of each CT scanner/acquisition protocol, in order to optimize CT imaging procedures.
